# Elastic energy storage in seahorses leads to a unique suction flow dynamics compared with other actinopterygians

**DOI:** 10.1242/jeb.236430

**Published:** 2021-09-03

**Authors:** Corrine Avidan, Roi Holzman

**Affiliations:** 1 School of Zoology, Faculty of Life Sciences, Tel Aviv University, Tel Aviv 69978, Israel; 2 The Inter-University Institute for Marine Sciences, PO Box 469, Eilat 88103, Israel

**Keywords:** LaMSA, Kinematics, Syngnathidae, Fish

## Abstract

Suction feeding is a dominant prey-capture strategy across actinopterygians, consisting of a rapid expansion of the mouth cavity that drives a flow of water containing the prey into the mouth. Suction feeding is a power-hungry behavior, involving the actuation of cranial muscles as well as the anterior third of the fish's swimming muscles. Seahorses, which have reduced swimming muscles, evolved a unique mechanism for elastic energy storage that powers their suction flows. This mechanism allows seahorses to achieve head rotation speeds that are 50 times faster than those of fish lacking such a mechanism. However, it is unclear how the dynamics of suction flows in seahorses differ from the conserved pattern observed across other actinopterygians, or how differences in snout length across seahorses affect these flows. Using flow visualization experiments, we show that seahorses generate suction flows that are 8 times faster than those of similar-sized fish, and that the temporal patterns of cranial kinematics and suction flows in seahorses differ from the conserved pattern observed across other actinopterygians. However, the spatial patterns retain the conserved actinopterygian characteristics, where suction flows impact a radially symmetric region of ∼1 gape diameter outside the mouth. Within seahorses, increases in snout length were associated with slower suction flows and faster head rotation speeds, resulting in a trade-off between pivot feeding and suction feeding. Overall, this study shows how the unique cranial kinematics in seahorses are manifested in their suction-feeding performance, and highlights the trade-offs associated with their unique morphology and mechanics.

## Abstract (precis)

**Highlighted Article:** Seahorses generate high suction flow and head rotation speeds with temporal patterns that differ from those of other actinopterygians; variation in snout length results in a trade-off between pivot and suction feeding.

## INTRODUCTION

Suction feeding is a highly stereotypic feeding behavior employed by the majority of aquatic vertebrates, including fishes, amphibians, reptiles, birds and mammals (Bardet et al., 2013; Cundall et al., 1987; Enstipp et al., 2018; Jacobs and Holzman, 2018; Marshall et al., 2008; Muller and Osse, 1984; Werth, 2005). This feeding behavior involves the predator closing the distance to the prey, rapidly opening its mouth and expanding the buccal cavity in order to draw water and the prey into their mouth. The buccal cavity is expanded by the rotation of the hyoid bone and the elevation of the head, powered by the coordinated shortening of the hypaxial and epaxial muscles, respectively. Water is drawn into the expanding mouth and evacuated through the gill slits, whose covers (the opercula) open through the shortening of the opercular dilator muscle as well as the levator operculi muscles, which contract in a lateral downward direction (Camp et al., 2015, 2018; Wainwright et al., 2007). Within fishes, the mechanisms that drive suction feeding are phylogenetically conserved (i.e. reflecting the tendency of fish species to retain their ancestral traits; Kraft et al., 2007).

These conserved mechanics also lead to conserved suction dynamics (Day et al., 2015). Suction-feeding kinematics follow an anterior-to posterior wave of expansion in which the mouth, buccal cavity and gill chamber open sequentially (Bishop et al., 2008). The wave of expansion generates a unidirectional water flow at the mouth, and peak flow speeds that coincide with peak gape and peak head elevation. Across teleost species, 85% of the inter-species variation in peak flow speed is explained by gape size and the time it takes the fish to open its mouth (Jacobs and Holzman, 2018). The spatial pattern of suction flow is also highly conserved, with the water drawn from a mushroom-shaped volume in front of the mouth. Within that volume, the speed of the flow decays rapidly, such that at a distance of 1 mouth diameter away from the center, flow speed is ∼10% of the speed at the mouth center (Day et al., 2015; Jacobs and Holzman, 2018; Muller et al., 1982). These conserved patterns are shared among fish from different trophic niches, habitats and evolutionary histories (Jacobs and Holzman, 2018).

Even against the background of considerable morphological and functional diversity of teleosts, seahorses stand out as extreme. They feature the distinctive vertical posture that inspired their name, reduced fins (Blake, 1976), shallow eye sockets (Mosk, 2004), a long snout and square ring-like segments in the axial skeleton (Porter et al., 2015). Within the Syngnathiformes (Longo et al., 2019; Van Wassenbergh et al., 2008), the order containing seahorses, pipefishes and snipefishes, a unique system has evolved to store energy from contracting muscles and release it abruptly in order to power their suction flows (Avidan et al., 2020 preprint) as well as an extreme rotational elevation of the head (pivot feeding; Longo et al., 2018; Van Wassenbergh et al., 2008). These coordinated functions are used to rapidly close the distance to the prey and suck it into the mouth. In sea dragons and snipefishes, these functions are powered only by the epaxial tendons (Longo et al., 2018; Stiller et al., 2015), while in seahorses the system is actuated by two tendons, the epaxial and the sternohyoideus (Avidan et al., 2020 preprint; Roos et al., 2009a). This dual latch-mediated spring actuated (LaMSA) system in seahorses drives head elevation speeds that are an order of magnitude faster than those of non-LaMSA teleosts. For example, time to peak gape is ∼2 ms in seahorses compared with 10–60 ms in non-LaMSA teleosts, and the angular speed of head elevation in seahorses can reach 10,000 deg s^−1^, compared with 350 deg s^−1^ for other teleosts. Unlike in other fishes, in seahorses there is a discord between lateral buccal expansion and hyoid depression, with hyoid depression preceding lateral head expansion (Roos et al., 2009b). Thus, it is unclear whether the magnitude and dynamics of these LaMSA-assisted suction flows differs from the conserved patterns observed across teleosts.

Snout length is a major axis of morphological diversity within seahorses. The pygmy seahorse, *Hippocampus bargibanti*, has an extremely short snout (22% of head length) while the spiny seahorse, *Hippocampus histrix*, has the longest snout (55% of head length) (Lourie et al., 2004). Importantly, the snout length of seahorses has several effects on feeding dynamics, possibly driving a trade-off between suction-feeding and pivot-feeding performance. A dynamic model of pivot feeding (de Lussanet and Muller, 2007) as well as a computational fluid dynamics (CFD) model of the expanding mouth cavity in seahorses (Roos et al., 2011) predicted that the time to reach the prey would diminish with increasing snout length and with decreasing gape diameter. These predictions were supported by a comparison of the feeding kinematics of two pipefish species with different snout lengths, which indicated that a longer snout is associated with faster angular speeds of head rotation, as well as faster displacement of the mouth (van Wassenbergh et al., 2011). Conversely, the ontogeny of feeding kinematics in the longsnout seahorse, *Hippocampus reidi*, indicated that the angular head rotation velocity decreased with age, corresponding to the elongation of the snout. Additionally, time to maximal rotation increased with age despite a decrease in the range of motion (maximal angle) (Roos et al., 2011). This difference from model predictions might be due to variation in the input force that drives head elevation, which could not be determined in the observational studies. In addition to their effect on pivot-feeding performance, snout length and mouth diameter are also expected to affect suction-feeding performance. Numerical simulations (Roos et al., 2011) predicted that suction volume and maximal flow velocity should increase with decreasing snout length, while the time to reach the prey via cranial rotation would increase. However, our understanding of suction-feeding dynamics and performance within seahorses is based on modeling and simulations, with no experimental measurements of these dynamics or of the expected trade-off between suction feeding and pivot feeding performance.

The aim of this study was to experimentally characterize suction-feeding performance in LaMSA-powered seahorses. We used a flow visualization setup to characterize the hydrodynamics and kinematics in three species of suction-feeding seahorses, with snout lengths ranging from 4.5 to 9 mm in length. Using these data, we (1) compared the magnitude and the spatio-temporal patterns of suction flows in seahorses with those of non-LaMSA fishes; and (2) tested the hypothesis that peak flow speed and head rotation speed trade-off within seahorses as a result of the contrasting effects of snout length on these performances.

## MATERIALS AND METHODS

### Study organism

**Fig. 1.**
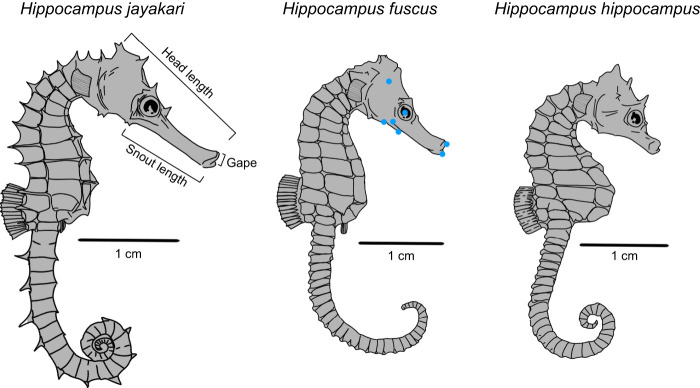
**Cranial morphology constitutes a major axis of morphological diversification in seahorses, as evident in the three species studied.** Head length is defined as the distance from the midpoint of the cleithral ring to the tip of the upper jaw; snout length is defined as the distance from the hyoid joint to the tip of the lower jaw. Blue points on the skull of *Hippocampus fuscus* denote the landmarks digitized to track head elevation, hyoid bone depression and gape-opening kinematics. Illustrations are to scale.

We focused on three seahorse species: *Hippocampus jayakari* Boulenger 1900 (*n*=3 individuals), *Hippocampus fuscus* Rüppell 1838 (*n*=2 individuals) and *Hippocampus hippocampus* (Linnaeus 1758) (*n*=4 individuals) (Fig. 1). The species differed in their snout length and gape size, with *H. hippocampus* having the shortest snout length (range 4.8–5.7 mm) and *H. jayakari* having the longest snout length (range 8.4–9.04 mm). The adult individuals from the different species had similar body sizes (standard lengths of 12.2, 10.0 and 11.4 cm for *H. jayakari*, *H. fuscus* and *H. hippocampus,* respectively). In addition, we filmed four *H. jayakari* juveniles (age 7–10 weeks) with snout lengths ranging from 5.3 to 7.0 mm. All individuals studied were obtained through the aquarium trade and kept in aerated 30 l seawater tanks for the duration of the experiments and all individuals were fed mysids. Animal care guidelines and regulations strictly followed the IACUC approved guidelines at the Hebrew University in Jerusalem, which oversees the experiments at the Inter-University Institute in Eilat.

### Pivot-feeding kinematics

The dynamics of the suction flows and the kinematics of pivot feeding were jointly visualized using a high-speed video camera and a solid state continuous wave laser that provided illumination for filming. Fish were filmed with a Photron SA3 2000 high-speed video camera (Photron, Tokyo, Japan) operating at 4000 Hz, at a resolution of 512×512 pixels. The camera was equipped with a 105 mm Nikon lens (*f*=2.8, Nikon, Tokyo, Japan). The laser (Magnum II Laser, 680 nm, 1.2 W, 10 deg fan angle; Coherent, Santa Clara, CA, USA) was equipped with a built-in optical system that created a vertical light sheet, <0.5 mm thick and ∼5 cm high. To obtain lateral views of pivot-feeding strikes, the camera was positioned orthogonally to the light sheet, with prey suspended on a thin wire inside the overlapping light sheet and field of view of the camera. The seahorses were trained to approach the prey from a perch position within the laser sheet to ensure that the light sheet was aligned with the sagittal plane of the head (Movies 1 and 2). In order to verify the position of the seahorse within the light sheet, a commercial video camera (GoPro Hero4, GoPro Inc., San Mateo, CA, USA) was located above the aquarium and only sequences in which the light sheet bisected the seahorse snout were used for this study. This alignment of the light sheet and the mouth was easy to gauge as consequence of the elongated snout, and because the fish were stagnant before abruptly moving their head upwards to feed. For each individual, at least 15 prey-capture events in which the sagittal plane of the seahorse was perpendicular to the symmetry axis of the camera lens were analyzed. Overall, 282 feeding strikes were analyzed for our three study species.

**Fig. 2.**
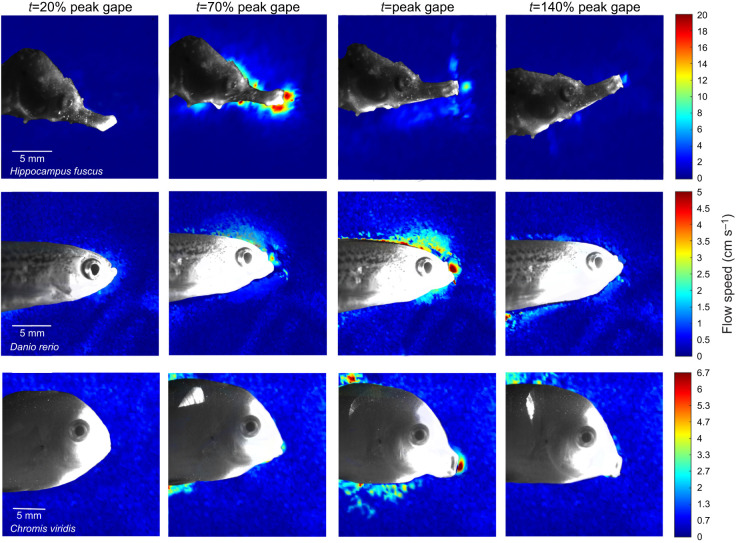
**Quantification and visualization of suction flow in front of the mouth during prey-capture strikes.** Depicted are false color images from particle image velocimetry (PIV) of flow speeds at different time points during the strike for a seahorse with a dual latch-mediated spring actuated (LaMSA) system and two non-LaMSA teleost species. Warmer colors represent faster flows, blue represents slow flows. Note that different scales were used for the flow speed in seahorses (0–20 cm s^−1^) and the two other species (0–5 and 0–6.7 cm s^−1^). Time to peak gape was 4.6±1.8, 25±15 and 40±20 ms for *H. fuscus*, *Danio rerio* and *Chromis viridis*, respectively. For all three species, suction flow impacts a small area in front of the mouth (Fig. 7), but the temporal sequence of the cranial events differs between the seahorse and the two other species (Figs 4 and 6).

For all species, the kinematic profile of each strike was determined through digitizing and tracking the anatomical landmarks of the proximal tip of the upper and lower jaw, the distal tip of the hyoid bone, the center of the eye socket and an anatomical landmark at the posterior end of the head, all clearly visible under the laser light sheet illumination (Figs 1 and 2). Tracking was done using the MATLAB function DLTdv5 (Hedrick, 2008). These landmarks enabled calculation of the following kinematic variables: (1) gape diameter, defined as the distance between the upper and lower jaw points; (2) hyoid depression distance, defined as the change in distance between the hyoid bone and the eye point; and (3) head rotation, defined as the change in head angle compared with its resting position before the strike. Head angle was defined as the angle, at the earthbound frame-of-reference, of the imaginary line connecting the center of the mouth and the landmark at the posterior end of the head. These variables were tracked through all high-speed video frames, enabling the calculation of: (1) time to peak gape (TTPG), defined as the time from when gape diameter first exceeded 20% of its maximal value to the time when it first exceeded 95% of its maximal value; and (2) time to peak hyoid displacement (TTHD), (3) time to peak head rotation (TTHR) and (4) time to peak flow speed (TTFS), all similarly calculated. We also determined for each strike the angular speed of head rotation, defined as the slope of the cumulative change in head rotation with respect to time.

**Fig. 3.**
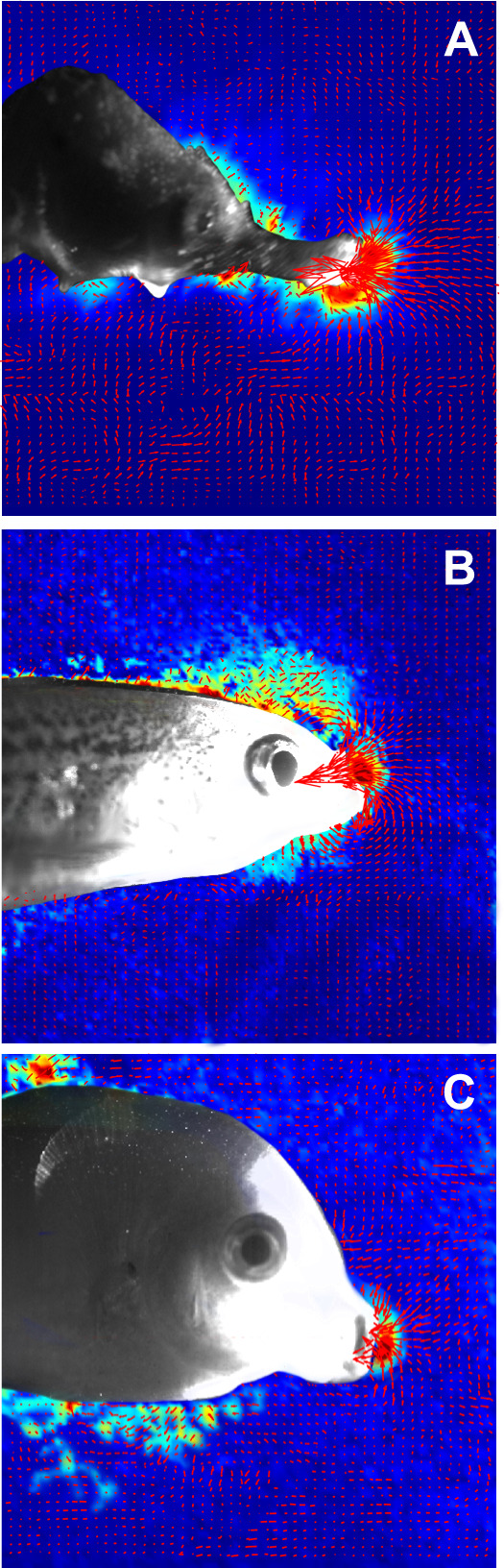
**Suction feeding at the time of peak suction flow.** Quiver images of (A) *H. fuscus* (B) *D. rerio* and (C) *C. viridis* showing both the direction and magnitude of flow, represented by red arrows.

**Fig. 4.**
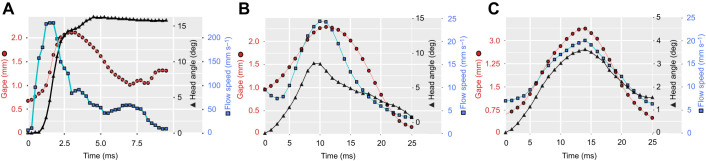
**Strike kinematics in seahorses and non-LaMSA fishes.** (A) In *H. fuscus*, peak flow speed precedes peak gape, and head angle does not return to its starting angle upon mouth closure. (B,C) In contrast, in non-LaMSA fishes (B, *D. rerio*; and C, *C. viridis*), the kinematics of gape diameter, head angle and flow speed follow a coordinated temporal pattern. Note that gape diameter is similar for the three species, but that both flow speed and time to peak excursion are an order of magnitude faster in *H. fuscus.* Note the different *x*- and *y*-axis scales.

**Fig. 5.**
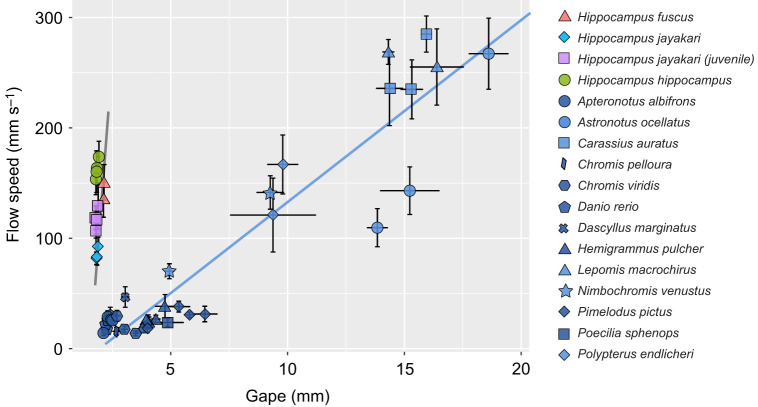
**Scaling of suction-feeding performance with gape diameter in non-LaMSA actinopterygians and dual-LaMSA seahorses.** Data are mean peak flow speed as a function of mean gape size for 50 individuals belonging to 16 species: 13 species of non-LaMSA actinopterygians (blue symbols) and 3 species of seahorses (red, turquoise, purple and green symbols; data for *Hippocampus jayakari* juveniles and adults are plotted separately). The scaling of peak flow speed with gape size is 8-fold steeper in seahorses compared with non-LaMSA actinopterygians. Error bars indicate standard error. Data for non-LaMSA actinopterygians are from Jacobs and Holzman (2018).

**Fig. 6.**
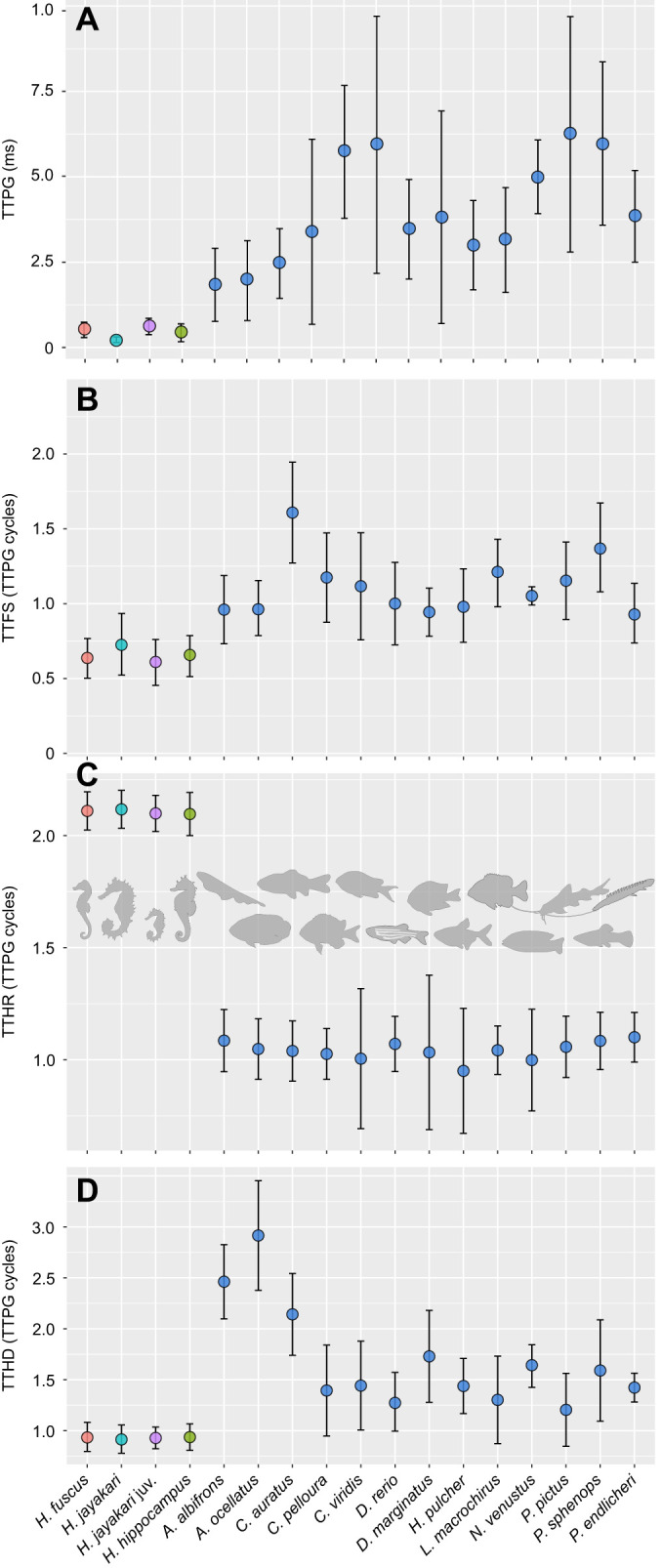
**Temporal pattern of suction feeding differs between non-LaMSA actinopterygians and dual-LaMSA seahorses.** Time to peak gape (TTPG; A) is significantly shorter in seahorses, and suction flow (time to peak flow speed, TTFS; B) and hyoid bone depression (time to peak hyoid displacement, TTHD; D) peak prior to peak gape in seahorses, whereas head rotation (time to peak head rotation, TTHR; C) is considerably delayed. Furthermore, seahorses have highly stereotypic timing, as indicated by the low standard errors of the timing variables. These trends are also evident in Figs 2 and 3. Data present species means for 50 individuals belonging to 16 species (see Fig. 5). Error bars depict standard error. Note that *y*-axis values in B–D are scaled to TTPG.

**Fig. 7.**
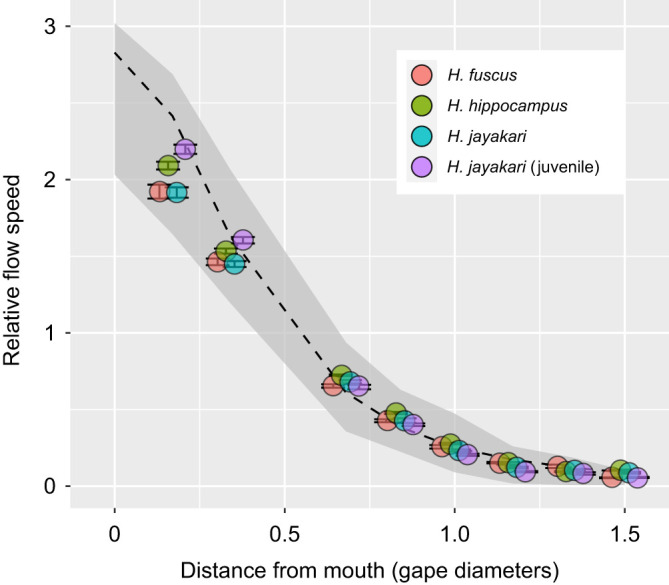
**Spatial pattern of suction flow around the mouth is similar between non-LaMSA actinopterygians and seahorses.** Data are the scaled flow speed (relative to the speed at half-gape distance from the mouth) versus the distance from the mouth (in units of gape diameter) for the 3 dual-LaMSA seahorse species (*n*=5 transects per species; ±standard error). The gray shaded area encompasses the range of observed data for 37 individuals belonging to 13 species of non-LaMSA actinopterygians (Jacobs and Holzman, 2018). The slope of decreasing flow speed with distance is statistically significant between seahorses and non-LaMSA actinopterygians (*P*<0.001); however, the effect size is small. The dashed black line represents the expected decay of flow speed based on Muller et al. (1982). Only data from at least 2 interrogation areas away from solid snout boundary were used for this analysis.

### Particle image velocimetry

More detailed doctrines of particle image velocimetry (PIV) can be found in Stamhuis (2006) and Taylor et al. (2014) and an abbreviated description is provided here. The water in the tank was seeded with 10 µm hollow glass spheres illuminated by a laser light sheet. A cross-correlation algorithm was implemented in MatPIV (Sveen, 2004), a freely available toolbox for analyzing PIV in MATLAB (The MathWorks, Natick, MA, USA). This algorithm used the displacement of the spheres between consecutive video frames to obtain estimates of the flow speed and direction at each location on a regularly spaced grid of 32×32 cells, with 50% overlap between adjacent cells (resulting in an estimated speed on a grid of 16×16 pixels; Fig. 2). The algorithm also calculated the signal-to-noise ratio (SNR) used to validate the velocity measurements. Verification and quality control of PIV results followed the protocols described in Stamhuis (2006) and Jacobs and Holzman (2018) and included inspection of SNR, rouge vectors, quiver plots and velocity profiles (see below). Throughout the text, we only discuss suction flows, and all of the velocities are 2D magnitudes with directionality towards the mouth.

The flow velocities created during suction feeding in front of the mouth were extracted along 13 transects extending from the center of the mouth anteriorly at different angles (Jacobs and Holzman, 2018). At 0 deg, the centerline transect protrudes from the center of the mouth forward, parallel to the snout, with 12 additional transects originating from the center of the mouth and extending in increments of ±10 deg from the centerline transect. To evaluate the spatial flow patterns, we extracted the flow speeds along each one of the 13 transects, at 10 locations along the transect (from mouth center to 1.5 gape diameters away), for an arbitrary frame in 5 PIV sequences for each individual. For each strike, we calculated the relative timing of peak flow speed, peak hyoid depression and peak head rotation. Because of variations in the timing of kinematic events across and within species, times were scaled to TTPG, i.e. a value of 1 denotes the temporal overlap with the time of peak gape (Fig. 6; Fig. S1). In order to ensure quality control during a feeding strike, velocities within 2 interrogation areas from the fish's body (boundary line set by intensity threshold and connected pixels) were excluded from analysis. This reduced the halo of scattered light from the solid surface of the fish to <10%, allowing a significant difference between background light and particle intensity. SNR values of all velocity vector analyses were >5, indicating a strong signal of particles relative to this scattered light. Vectors larger than 3 standard deviations from the local 3 values were removed. No interpolation was conducted, and no vectors were replaced. To characterize the temporal pattern of suction flows, we extracted, in each time frame, the flow speeds at a distance of 0.5 gape diameters from the mouth on the center line. From these data, we calculated peak flow speed in each PIV sequence. We used the distance of half-gape to facilitate comparison with previous studies (Day et al., 2005; Higham et al., 2006; Jacobs and Holzman, 2018).

### Statistical analyses

We used new data collected for seahorses (see above), as well as those previously published in Jacobs and Holzman (2018) for non-LaMSA fishes. Overall, we analyzed 660 strikes by 50 individuals belonging to 16 species. To compare the magnitude of suction flows in seahorses with non-LaMSA fishes we ran a mixed-effect linear model, with peak flow speed at half-gape as the dependent variable, peak gape size and the presence of a LaMSA system as fixed factors along with their interaction, and species as a random factor.

To examine the effect of LaMSA on the spatial pattern of flow speed decay, we ran a mixed-effect linear model with flow speed measurements at 10 distances from the mouth as the dependent variable, distance from the mouth and the presence of a LaMSA system as fixed factors along with their interaction, and species and individual as random factors. We used individual averages of 3–5 strikes per individual, with a total of 48 individuals of 16 species. Flows were measured at equally spaced intervals between 0 and 1.5 gape diameters away from the mouth; we excluded the distance of 0.5 gape diameters, where flow speed was scaled to 1. We also ran a second mixed-effect linear model with the same parameters as above with the addition of flow speed measurements from transects other than the center line. These transects extend from the mouth center outwards at 30 and 60 deg from the center line and were found to be indistinguishable from the speeds measured on the centerline (mixed-effect model, *F*_1,318_=15.4, *P*>0.05, whole model *P*<0.001).

To test the hypothesis that a LaMSA system affects the timing of kinematic events, we ran a series of mixed-effect models, with the timing variable (TTFS, TTHD, TTHR) as the dependent variable, the presence of LaMSA as an independent variable, and species as a random factor. We used individual averages of ∼15 strikes per individual, with a total of 48 individuals belonging to 16 species. Note that the analysis was run separately for each timing variable (TTFS, TTHD, TTHR). All mixed-effects models were run using the LMER package in R (Bates et al., 2015; http://www.R-project.org/).

## RESULTS

### Do suction flows differ between seahorses and non-LaMSA fishes?

Flow visualization experiments (Fig. 2) revealed that peak flow speed in seahorses was 8 times faster than that in non-LaMSA fishes with a comparable gape diameter (Figs 2–5), as indicated by the significant effect of LaMSA on the slope of peak flow speed as a function of peak gape (mixed-effect model, *F*_1,618_=137.9, *P*<0.001, whole model *P*<0.001; Table S1). For example, mean peak flow speed (±s.e.m.) for *H. hippocampus* was 162±4 mm s^−1^ (*n*=108) with a mean gape of 1.8 mm, while the mean peak flow speed for the zebrafish (*D. rerio*) was 20±10 mm s^−1^ (*n*=27) with a similar mean gape (1.9 mm; Figs 2–4; peak flow speed measured at a distance of 0.5 gape diameters away from the mouth center). Correspondingly, TTPG and TTFS were both significantly faster in seahorses, with a mean TTPG of 2.5±0.18 ms and a mean time to peak flow speed of 2.1±0.011 ms, versus a mean TTPG of 41 ms and a mean TTFS of 43 ms for non-LaMSA fishes (Fig. 6; mixed-effect model, *F*_1,14.8_=15.7, *P*<0.005 for TTPG and *F*_1,14.7_=19.1, *P*<0.001 for TTFS).

The temporal pattern of cranial events in seahorses differed from that observed in non-LaMSA fishes (Figs 2–6). Peak flow (normalized to TTPG) preceded peak gape by 20% in seahorses, whereas in non-LaMSA fishes peak flow coincided with peak gape (mixed-effect model, *F*_1,14.4_=14.0, *P*<0.005; Fig. 6B). Peak head rotation (normalized to TTPG) occurred much later than peak gape in seahorses (at 8.4±2.2 ms), whereas in non-LaMSA fishes peak head rotation coincided with peak gape (mixed-effect model, *F*_1,15.2_=33, *P*<0.001; Fig. 6C). TTHD did not significantly differ between seahorses and non-LaMSA fishes, occurring around 95% of TTPG (mixed-effect model, *F*_1,14.8_=14.8, *P*>0.5; Fig. 6D).

The spatial pattern of suction flows in seahorses was similar to that observed in non-LaMSA fishes. The slope of decaying flow speed with distance from the mouth was significantly different between the seahorses and non-LaMSA fishes (mixed-effect model, *F*_1,318_=15.5, *P*<0.005, whole model *P*<0.001; Table S2). However, the effect size of LaMSA on the speed of this decay was small (Fig. 7): the coefficient for the effect of distance on flow speed was −0.043 gape^−1^ for seahorses and −0.739 gape^−1^ for non-LaMSA fishes. Because the decay is exponential, this model predicts that flows equal to 10% of the flow at the center of the mouth will occur at a distance of 1.21 gape dimeters for seahorses and 1.1 gape dimeters for non-LaMSA fishes (a difference corresponding to an additional 0.2 mm for seahorses).

### Does snout morphology affect suction-feeding performance in seahorses?

**Fig. 8.**
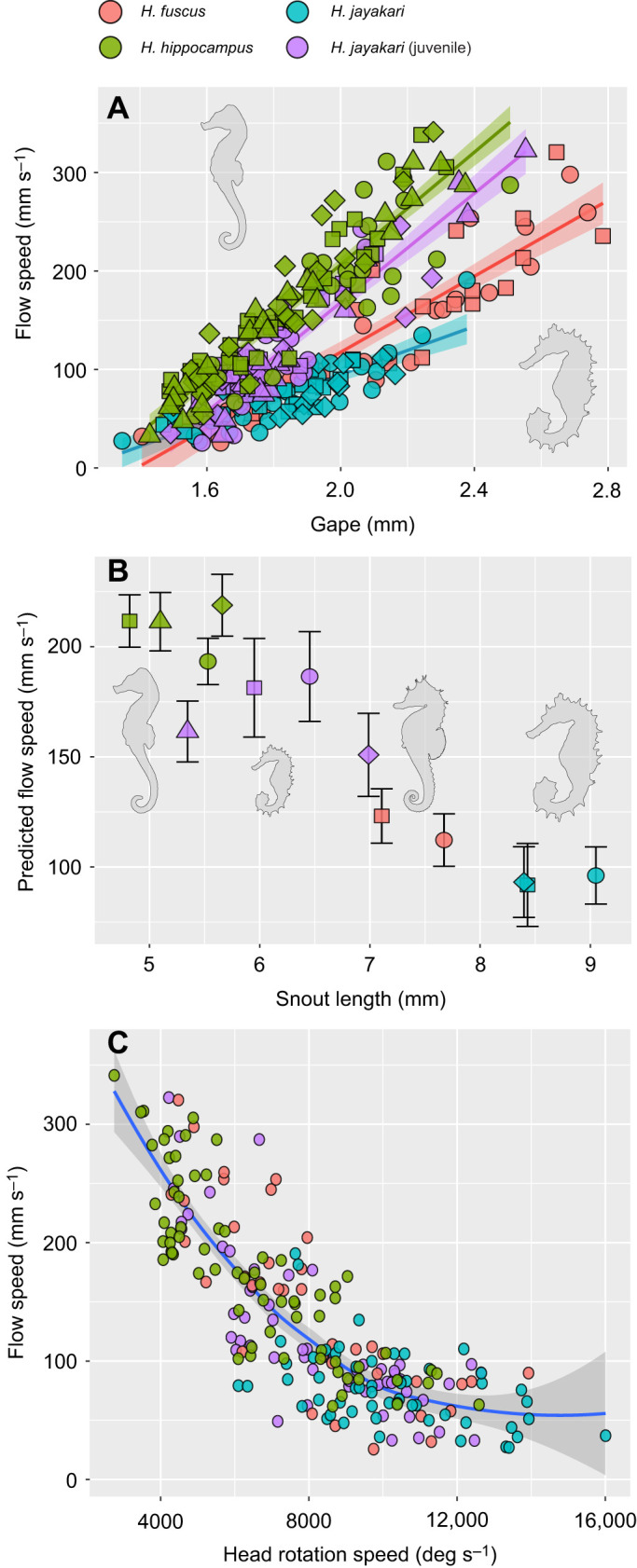
**Differences in snout length are manifested in a trade-off between pivot feeding and suction feeding in seahorses.** (A) The scaling of peak flow speed with gape diameter is shallower in long-snouted species (mixed-effect model *P*<0.001). Data are all strikes by all individuals. (B) The predicted flow speed, calculated from the mixed-effect model as the flow speed expected for each individual if gape is 2 mm, decreases with snout length. Data are individual means, error bars are standard error of the model's estimate. (C) Peak suction flow speed trades off with the speed of head rotation in seahorses. Data are all strikes by all individuals. The blue line represents the slope of a linear regression between flow speed and log-transformed head rotation speed. Gray shading represents the standard error of the estimated slope. Results are based of 282 PIV realizations of suction-feeding strikes by 13 individuals belonging to 3 species of seahorses. *Hippocampus jayakari* individuals were divided into juveniles and adults because of their different snout allometry and snout length.

Across the studied seahorse species, snout length was negatively correlated with the magnitude of peak flow speed (linear regression on individual means; *F*_1,11_=24.0, *P*<0.005, *R*^2^=0.69; Fig. 8A,B). *Hippocampus jayakari*, the species with the longest snout (mean 9.04 mm) had a mean peak flow speed of 0.08 m s^−1^ at a distance of 0.5 gape diameters away from the mouth, whereas *H. hippocampus* (4.8 mm snout) had a mean peak flow speed of 0.16 m s^−1^. The angular speed of head rotation showed the opposite relationship, increasing with snout length (linear regression on individual means; *F*_1,11_=47.8, *P*<0.001, *R*^2^=0.81). These trends resulted in a trade-off between the magnitude of suction flow and the angular speed of head rotation (linear regression on individual means; *F*_1,11_=171.2, *P*<0.001, *R*^2^=0.93; Fig. 8C). For example, *H. jayakari* had a mean angular speed of 10,197 deg s^−1^, whereas *H. hippocampus* had a mean angular speed of 6564 deg s^−1^.

## DISCUSSION

Seahorses use a unique power amplification dyad to power their coordinated pivot-feeding and suction-feeding behavior. This dual system allows seahorses to generate suction flows and head rotation speeds that are at least an order magnitude faster than those of similar sized fish that lack a LaMSA system (Figs 5 and 8). The presence of the LaMSA system alters the temporal patterns of suction flows, compared with the conserved pattern across other actinopterygians (Figs 2, 4 and 6; Jacobs and Holzman, 2018). However, the spatial patterns of suction flows remain constrained, impacting a radially symmetric region of ∼1 gape diameter outside the mouth (Fig. 2; see also Fig. 7). Differences in the magnitude of peak suction flow and head rotation speed between the three studied seahorse species were associated with differences in snout length (Fig. 8A,B), and manifested in a trade-off between pivot feeding and suction feeding (Fig. 8C). Altogether, our results demonstrate the performance envelope of LaMSA-powered suction feeding, and highlight the trade-offs and limitations associated with LaMSA-driven performance.

The presence of a LaMSA system dramatically improves both suction-feeding and pivot-feeding performance in seahorses, compared with non-LaMSA fishes. The faster head rotation, coupled with the long snout, enables seahorses to rapidly bring their mouth next to the prey within 2–4 ms, a time frame much shorter than is needed for evasive copepods to engage in an escape response. For example, *Acartia* spp. typically initiate their escape response within 4 ms of sensing a hydrodynamic disturbance, with multiple power strokes of their swimming legs, with each stroke lasting ∼7 ms and accelerating the copepods to speeds up to 500 mm s^−1^ (Buskey et al., 2002). The LaMSA-powered suction flow of seahorses complements the fast head rotation as it drags the prey into the mouth with an augmented force compared with non-LaMSA suction feeders. The suction force is amplified as a result of the combined effect of several hydrodynamic mechanisms. The force exerted on a prey within the suction flow is the result of three component forces: drag, acceleration reaction and pressure gradient force. Drag force is a function of flow speed squared, and is therefore expected to be steeply augmented in LaMSA-powered flows (Skorczewski et al., 2010; Wainwright and Day, 2007). The acceleration reaction force is a function of the spatial and temporal gradients in the flow (Figs 7 and 4, respectively). The latter gradients are enhanced in seahorses, because they generate faster flows in shorter time compared with non-LaMSA fishes, resulting in accelerations of 390 m s^−2^. Additionally, the rapid head rotation moves the area of high flow (in front of the mouth) towards the prey, such that flow speed at the location of the prey can change rapidly solely as a result of the movement of the mouth. This movement should have a similar effect to that of fast jaw protrusion (Holzman et al., 2008). Lastly, a smaller mouth will result in a steeper pressure gradient, which will augment the pressure gradient forces compared with those exerted by a larger mouth with the same flow speed (Wainwright and Day, 2007). Note that the presence of LaMSA had a minor effect on the spatial patterns of flow speed in front of the mouth. Similar to the finding in other actinopterygians, suction flows only impacted an area of approximately 1 gape diameter away from the mouth, and the flows adhered to a similar rate of decay as in other fish (Figs 2 and 7; Jacobs and Holzman, 2018).

The time to peak head elevation in seahorses was much later than either TTFS or TTPG. In our observations, seahorses intersected the prey between the time of peak flow speed and peak gape, while the head was moving rapidly upwards. In other actinopterygians, the plane of the mouth moves perpendicular to the plane's axes, such that the mouth will eventually engulf the prey if the latter does not move. In seahorses, the plane of the mouth moves parallel to the plane's vertical axis, resulting in a ‘drive-by’ motion. Consequently, the mouth would not engulf the prey even if the latter did not move. Given this movement, the high suction forces, produced by the seahorses, might be a necessary adaptation to draw the prey into the mouth before it moves past the prey.

While fast suction flows and head rotations are expected to be beneficial for prey capture, the kinematics of the cranial events could be interpreted as potential impediments. Unlike other actinopterygians whose suction flows peak with peak gape, seahorses reach peak flow speed partially through the gape cycle, before the gape is fully opened (Figs 4 and 6B). Thus, when the gape is fully opened and the reach of the flow is maximal, the flow speed has already subsided and is decelerating. In either case, seahorses cannot intercept their prey with the optimal combination of peak flow at peak gape (Bishop et al., 2008; Keren et al., 2018 preprint). This relative timing of peak flow and peak gape in seahorses is stereotypic (see low standard errors in all *Hippocampus* species in Fig. 6A,B), and is unrelated to strike effort (i.e. peak gape, TTPG or peak flow speed; linear regression *P*>0.05 and *R*^2^<0.05 for all). In other actinopterygians, a temporal gradient in muscle activation drives an anterior-to-posterior wave of buccal expansion that aligns peak flow speed and peak gape (Bishop et al., 2008; Carroll, 2004). It is possible that the deviation from this pattern in seahorses results from the mechanical constraints of the LaMSA system, in particular the transmission of motion through ligaments that connect the pectoral girdle, the hyoid and the lower jaw (Longo et al., 2018). This mechanical coupling could be the reason for the low standard errors found for all timing variables in seahorses (Fig. 6), compared with the larger variation found for other actinopterygians. However, this could also result from the difference in the relative sampling density for LaMSA and non-LaMSA species, i.e. the lower number of video frames that fit in a gape cycle of a seahorse could artificially reduce the variance between measurements.

Here, we add empirical measurements of suction feeding in seahorses to the previously published studies which examine the kinematics of head rotation as well as models of their suction feeding. While corroborating the presumed trade-off between suction flow speed and head rotation speed, our measurements illuminated some inconsistencies with the previous research. Previous studies reported that ventral and lateral expansion of the seahorse mouth cavity do not occur simultaneously, and concluded that ventral expansion would have the most dramatic effect on suction feeding (Roos et al., 2009b, 2011; Van Wassenbergh et al., 2013). As result of this assumption, previous models of suction feeding in seahorses assumed negligible suction flows during the head rotation process. Our measurements, however, clearly show that suction feeding follows hyoid kinematics (Fig. 6B,D), beginning as the mouth starts to open, peaking before peak gape, and subsiding as the head reaches peak elevation (Figs 2, 4 and 6). The results indicate that hyoid displacement might play a more important role in buccal expansion than previously understood. Furthermore, because the mass of the head increases as it is accelerated upwards, calculations of the power needed to realize head elevation (Van Wassenbergh et al., 2013) may be inaccurate.

Snout length is a major axis of morphological diversification in seahorses. Roos et al. (2011) used numerical simulations to evaluate both suction-feeding and pivot-feeding performances for seahorses with different snout lengths and gape diameters, in both juvenile and adult seahorses. Additional simulations accounting for the power needed to accelerate the rotating snout explored the expected kinematics of cranial events throughout seahorse ontogeny (Roos et al., 2011). These simulations yielded several predictions regarding the effects of snout length on performance. Species with a longer snout were expected to demonstrate faster head rotation, although head rotation speed was expected to decay throughout ontogeny. The simulations also predicted that the speed of suction flow would decrease with increasing snout length, resulting in an expected trade-off between suction feeding and pivot feeding. Our results corroborate the prediction regarding a trade-off between head rotation speed and suction flow speed driven by snout length (Fig. 8). However, we did not find support for the expected trend of decreasing head rotation speed with ontogeny, over the range of ages and snout lengths tested (Fig. 8C). Across our *H. jayakari* individuals (*n*=7), mean head rotation speed increased from 7900 deg s^−1^ for the individual with the shortest snout (snout length 5.3 mm) to 10,300 deg s^−1^ for the individual with the longest snout (snout length 9.04 mm).

We hypothesize that the performance trade-off associated with snout length would manifest in the diets and ecologies of long- and short-snouted seahorses. The short snout may be more suitable for more camouflaged seahorses that feed on visually guided prey, which typically have slower escape responses compared with prey that respond to hydrodynamic cues (hereafter ‘strain-sensitive’ prey; Buskey et al., 2002). The shorter snout would still move fast enough to intercept such prey, and would be beneficial to overcome stronger escape forces or draw larger prey into the mouth. The long snout species are expected to produce slower suction flows, but have the advantage of faster head rotation speed, and therefore might be more suitable for strain-sensitive prey that need to be overtaken before the escape response is initiated. A narrow profile of the snout can also contribute to minimizing the hydrodynamic signal available for the prey (Gemmell et al., 2013; Leysen et al., 2011). Indeed, a comparative study of 12 seahorse and pipefish species revealed that syngnathids characterized by long snouts consume highly mobile prey that are in constant motion, while relatively slower moving prey are favored for shorter-snouted species (Howard and Koehn, 1985; Kendrick and Hyndes, 2005). These conclusions are supported by previously published syngnathid diets, although comparisons are limited because of differences in dietary analyses. These results suggest that the functional demands of prey capture by both suction feeding and pivot feeding are driving the morphological variations seen in Syngnathidae.

We conjecture that our results are general within power-amplified Syngnathiformes, despite the fact that seahorses uniquely possess a dual LaMSA system, consisting of two elastic tendons (the sternohyoidius and the epaxial tendons). However, other power-amplified Syngnathiformes species appear to share a similar four-bar lever system which transmits power from the epaxial tendon to the rotating hyoid and head. We therefore expect the temporal patterns of suction flow and cranial kinematics in other power-amplified Syngnathiformes will be more similar to those of seahorses than to those of non-LaMSA actinopterygians. The spatial patterns of suction flow are highly conserved across all actinopterygians, including seahorses, and are unlikely to differ in other power-amplified Syngnathiformes. However, we expect the magnitude of flow speed and speed of head rotation to be higher in seahorses than in other power-amplified Syngnathiformes (Longo et al., 2018; van Wassenbergh et al., 2011), owing to the effects of the additional ligament in their LaMSA system. This transition between muscle-actuated and dual-spring actuated systems observed across Syngnathiformes offers a promising opportunity to study the evolution of LaMSA mechanics and its effects on suction feeding, pivot feeding and their interactions.

## Supplementary Material

Supplementary information
