# Supplementary material for: Elastic energy storage in seahorses leads to a unique suction flow dynamics compared with other actinopterygians
Source: J Exp Biol. 2021 Sep 3;224(17):jeb236430. doi: 10.1242/jeb.236430 (PMC8443865; doi:10.1242/jeb.236430)
Supplement: Supplementary information [file jexbio-224-236430-s1.pdf]

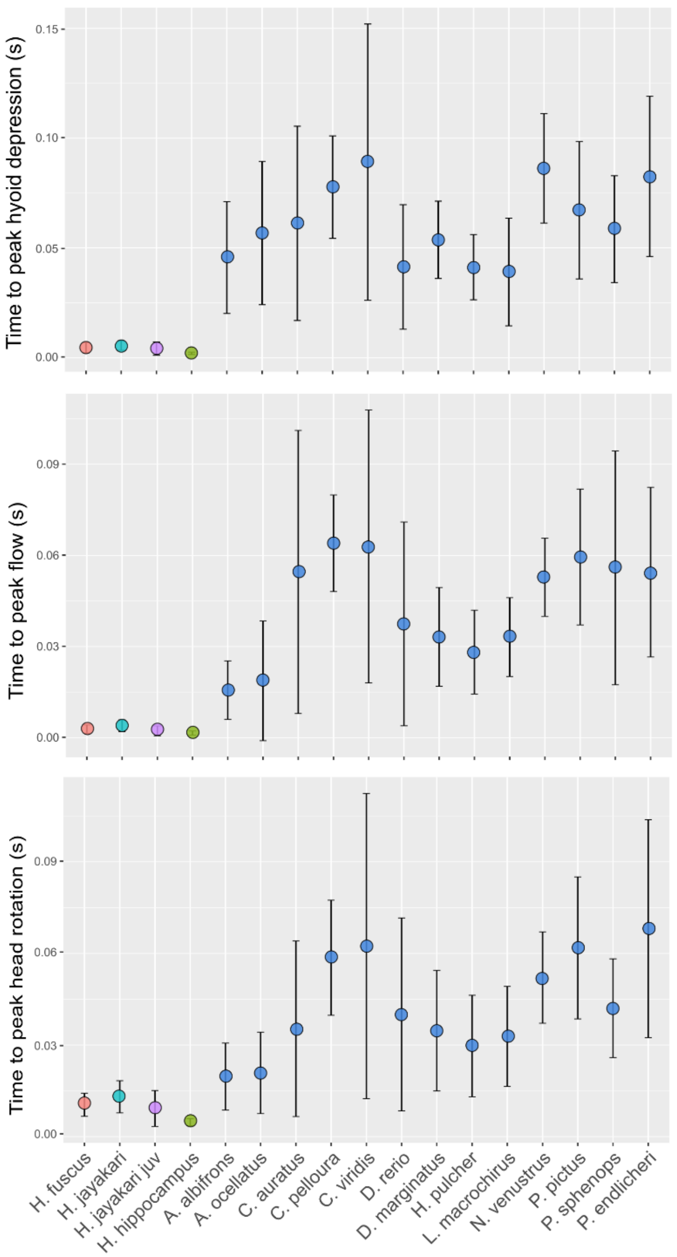

Fig. S1. Species averaged timing of kinematic events during suction feeding showing the differing temporal patterns between non-LaMSA actinopterygians (blue symbols) and dual LaMSA system in seahorses (red, turquoise, purple, and green symbols). All timings of events are significantly shorter in seahorses. (A) Time to peak hyoid depression (B) time to peak flow and (C) time to peak head rotation represent the major events during a feeding strike. Data present species means, for 50 individuals belonging to 16 species. Full species names are provided in Table S2. Error bars depict standard errors.

Journal of Experimental Biology • Supplementary information

Table S1. The coefficients (and their standard errors) from the Mixed-effect model featuring the effect of species, gape size and presence of LaMSA system on flow speed.

Intercept	LaMSA	Peak Gape	LaMSA: Peak Gape
49.26 ± 57.63	-547.68 ± 64.78	24.91 ± 1.04	89.78 ± 7.65

Table S2. The coefficients of both fixed (upper row) and random effects (bottom rows) from the Mixed-effect model featuring the effect of LaMSA, the distance from the mouth, and their interaction (LaMSA: Distance from mouth) on flow decay.

Intercept	LaMSA	Distance from mouth	LaMSA: Distance from mouth
1.39 ± 0.011	-0.043 ± 0.020	-0.739 ± 0.010	0.068 ± 0.017

Species	Intercept
Astronotus ocellatus	0
Apteronotus albifrons	0.0093
Carassius auratus	-0.0128
Chromis pelloura	0.0141
Chromis viridis	0.0235
Danio rerio	0.0152
Dascyllus marginatus	-0.0153
Hemigrammus pulcher	-0.0104
Hippocampus fuscus	-0.0002
Hippocampus hippocampus	0.0112
Hippocampus jayakari	-0.0013
Hippocampus jayakari fry	-0.010
Lepomis macrochirus	0.010
Nimbochromis venustus	-0.033
Pimelodus pictus	-0.008
Poecilia sphenops	0.0002
Polypterus endlicheri	0.0136

Journal of Experimental Biology • Supplementary information

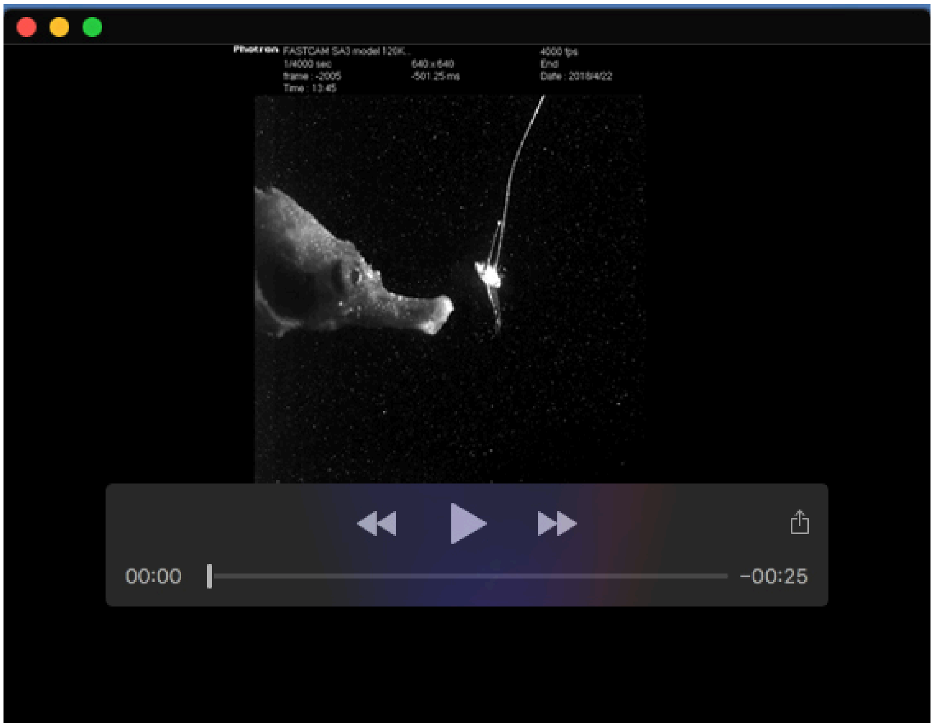

Movie 1. A suction feeding strike by H. hippocampus with the water flow visualized by particle imaging velocimetry.

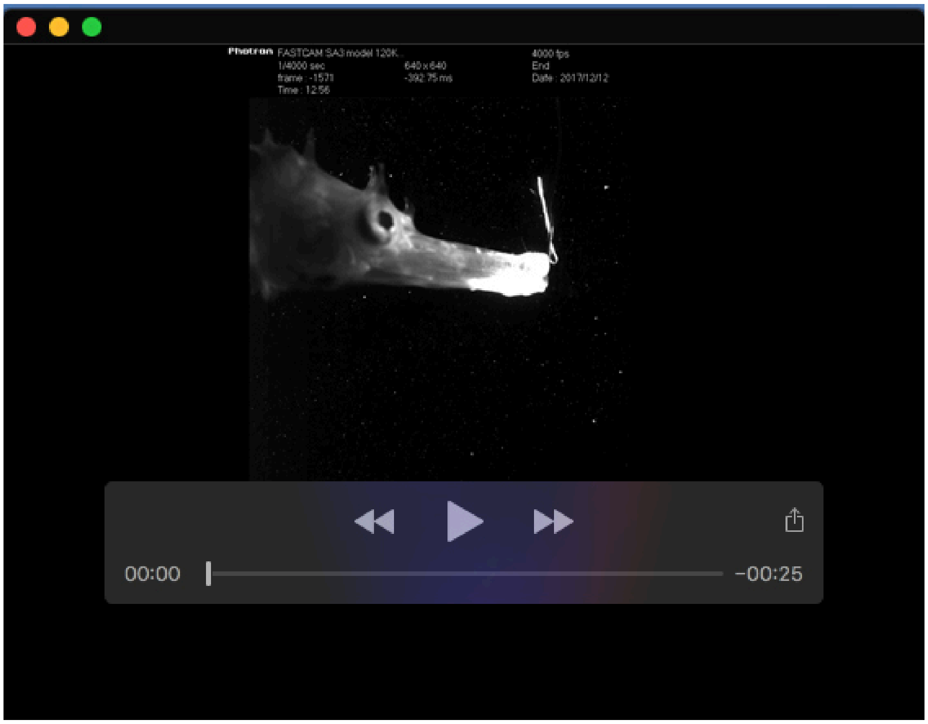

Movie 2. A suction feeding strike by H. jayakari with the water flow visualized by particle imaging velocimetry.
